# Inactive enzymatic mutant proteins (phosphoglycerate mutase and enolase) as sugar binders for ribulose-1,5-bisphosphate regeneration reactors

**DOI:** 10.1186/1475-2859-4-5

**Published:** 2005-02-02

**Authors:** Debojyoti De, Debajyoti Dutta, Moloy Kundu, Sourav Mahato, Marc T Schiavone, Surabhi Chaudhuri, Ashok Giri, Vidya Gupta, Sanjoy K Bhattacharya

**Affiliations:** 1 Department of Biotechnology, Haldia Institute of Technology, Haldia, West Bengal, India; 2 Environmental Biotechnology Division, ABRD Company LLC, 1555 Wood Road, Cleveland, Ohio, 44121, USA; 3 Plant Molecular Biology Division, National Chemical Laboratory, Pune 411008, India; 4 Department of Ophthalmic Research, Cleveland Clinic Foundation, Area I31, 9500 Euclid Avenue, Cleveland, Ohio, 44195, USA

## Abstract

**Background:**

Carbon dioxide fixation bioprocess in reactors necessitates recycling of D-ribulose1,5-bisphosphate (RuBP) for continuous operation. A radically new close loop of RuBP regenerating reactor design has been proposed that will harbor enzyme-complexes instead of purified enzymes. These reactors will need binders enabling selective capture and release of sugar and intermediate metabolites enabling specific conversions during regeneration. In the current manuscript we describe properties of proteins that will act as potential binders in RuBP regeneration reactors.

**Results:**

We demonstrate specific binding of 3-phosphoglycerate (3PGA) and 3-phosphoglyceraldehyde (3PGAL) from sugar mixtures by inactive mutant of yeast enzymes phosphoglycerate mutase and enolase. The reversibility in binding with respect to pH and EDTA has also been shown. No chemical conversion of incubated sugars or sugar intermediate metabolites were found by the inactive enzymatic proteins. The dissociation constants for sugar metabolites are in the micromolar range, both proteins showed lower dissociation constant (Kd) for 3-phosphoglycerate (655–796 μM) compared to 3-phosphoglyceraldehyde (822–966 μM) indicating higher affinity for 3PGA. The proteins did not show binding to glucose, sucrose or fructose within the sensitivity limits of detection. Phosphoglycerate mutase showed slightly lower stability on repeated use than enolase mutants.

**Conclusions:**

The sugar and their intermediate metabolite binders may have a useful role in RuBP regeneration reactors. The reversibility of binding with respect to changes in physicochemical factors and stability when subjected to repeated changes in these conditions are expected to make the mutant proteins candidates for *in-situ *removal of sugar intermediate metabolites for forward driving of specific reactions in enzyme-complex reactors.

## Background

One of the potential use of sugar and sugar intermediate binding proteins and binders of intermediate sugar metabolites derived from microbes is in new and expanding area of environmental biotechnology particularly in carbon dioxide fixation bioprocess reactors [1,2]. Accelerated consumption of fossil fuels and other anthropogenic activities have resulted in increased atmospheric levels of the greenhouse gas carbon dioxide (CO_2_). Sustained increase of atmospheric CO_2 _has already initiated a chain of events with unintended ecological consequences [3-7]. The reduction in atmospheric carbon dioxide level is highly desirable lest it will have a catastrophic impact upon both the environment and the economy on a global scale [5-7].

Biotechnological process with recombinant catalytic proteins offer contained handling of carbon dioxide and could be one method of abatement of carbon dioxide pollution [8,9]. Recent advances in biotechnological methods makes possible efficient capture [10] and fixation of CO_2 _at emission source/site into concatenated carbon compounds [9,11]. Such a process starts with initial capture of the carbon dioxide solublized as carbonic acid or bicarbonate [10]. After adjustment of pH using controllers and pH-stat the solution is fed to immobilized Rubisco reactors [12] where acceptor D-Ribulose-1,5-bisphosphate (RuBP) after CO_2 _fixation is converted into 3-phosphoglycerate [8,9]. We have invented a novel scheme which proceeds with no loss of CO_2 _(unlike cellular biochemical systems) in 11 steps in a series of bioreactors [13].

For starting up the process, however, a different scheme was used to generate RuBP from D-glucose rather than from 3-PGA [14]. The linear combination of reactors in the 11 step RuBP regeneration process requires large volume and weight and are unsuitable for use in mobile CO_2 _emitters leaving only the stationary source of emission to be controlled using this technology [8,9]. These problems are circumvented in a new scheme where enzyme-complex reactors instead of linear combination of purified single enzyme reactors were proposed [1,2]. In this scheme, the catalytic enzymes have been used as functionally interacting complexes/interactomes.

**Figure 1:**
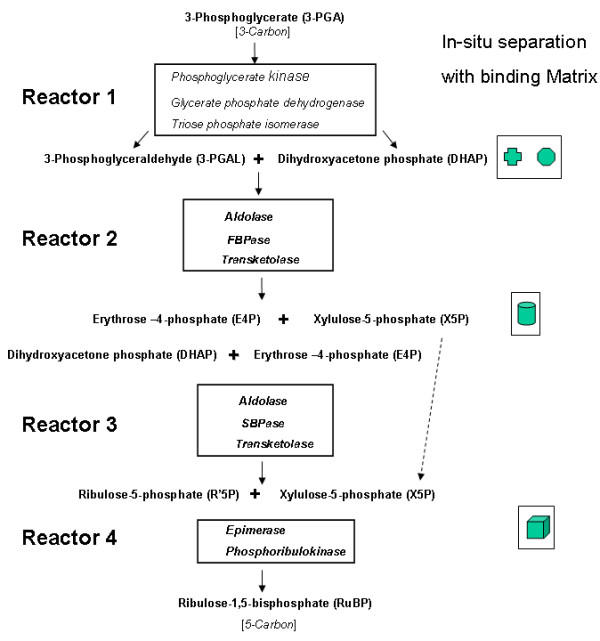
Arrangement of enzymes in four reactors with indicated enzyme complexes enabling internal channeling, greatly reduces volume and weight for regenerating reactors with faster overall conversion rate to RuBP starting with 3PGA making the system compatible for application in mobile devices in addition to stationary emitters [2]. The reactors may use the sugar binding entities at indicated positions, the solid symbols represent metabolite-bound matrix (binding protein entities), the plus, circle, cylinder and box are symbols for 3PGA, DHAP, X5P and RuBP binders respectively.

The four reactors harboring enzymatic complexes/mixtures [2] replace the current 11 reactors for conversion of 3PGA into RuBP [8,9] and are termed as enzyme-complex reactors (Figure 1). As an alternate to immobilized enzyme-complexes, in this scheme, successive conversion in radial flow with layers of single and purified but uniformly oriented enzymes in concentric circle with axial collection flow system with required combination of enzymes in individual reactors could also be used [1]. This arrangement in preliminary experiments shows promise and leads to a faster conversion rate and requires less volume and material weight. Sugar and sugar intermediate metabolite removal in enzyme-complex reactors at key steps is necessary for proper and specific driving of forward reactions. This requires in-situ separation of sugar or intermediate metabolites by specific binding entities. The compounds such as 3-phosphoglycerate (3PGA) and 3-phosphoglyceraldehyde (3PGAL) must be separated at key steps for proper functioning of these enzyme-complex reactors [1,2].

Sugar and their intermediate metabolites binding proteins derived from microbial and other sources despite being used for various applications [15,16] have not yet been used in environmental biotechnological applications. However, they are potentially applicable in RuBP recycling. In this report we demonstrate the utility of two inactive mutants of enzymatic proteins: phosphoglycerate mutase (PGDM) and enolase. The inactive mutants of yeast enzymes PGDM [17-19] and enolase [20,21] were characterized for properties that may render them potentially useful in reactors. We report determination of the enzymatic activity, sugar binding capacity, specificity in binding for different sugar and their metabolites and reversibility in binding with respect to changes in physicochemical factors and stability on repeatedly subjecting to these changes using purified proteins.

## Results

### Purity of proteins

**Figure 2:**
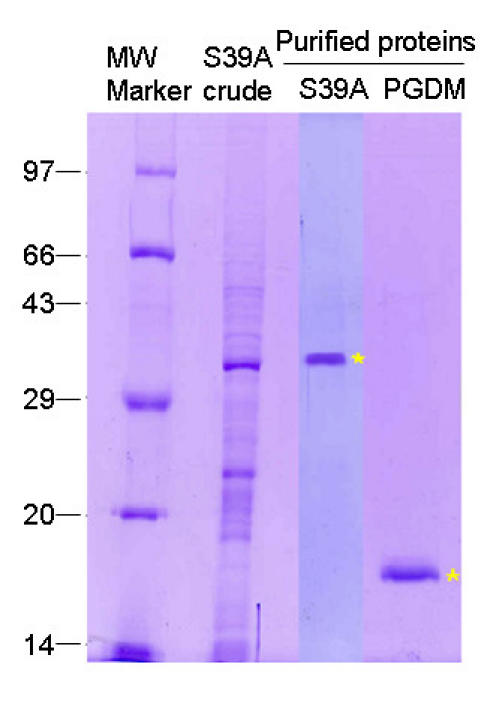
The crude lysate and purified proteins. About 5–10 μg of proteins were loaded on a 10% SDS-PAGE as indicated. The inactive PGDM and mutant yeast enolase 1 S39A and H159A has been indicated. The gel was stained with Coomassie blue.

**Figure 3:**
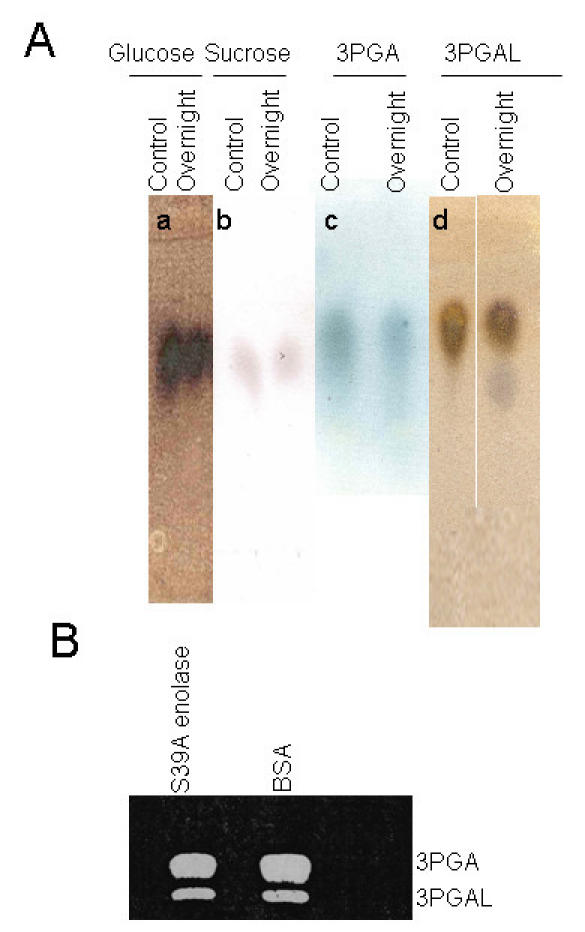
**A. **Representative paper chromatographic detection of sugars metabolites. The sugars or intermediate metabolites (0.1–0.5 mM) were incubated with BSA (control), PGDM or enolase mutants overnight at room temperature, proteins removed by centrifugation at 10000 × g and an aliquot of supernatant was spotted. a. d. The chromatogram for enolase mutant S39A stained with silver nitrate reagent, b. c. chromatogram for enolase mutant S39A stained with ammonium molybdate reagent. **B. **Representative thin layer chromatography of sugar metabolites (3-phosphoglycerate, 3-phosphoglyceraldehdye). About 0.1 mM substrate (0.1 mM of each mixture component) as indicated was incubated overnight with purified S39A enolase or BSA (control) has been shown. The chromatogram was developed using silver nitrate reagent following protocol as described in methods and image has been converted to greyscale.

The inactive PGDM mutant [17,18] was received as purified protein and analyzed on a 10% SDS-PAGE (Figure 2). The S39A mutant of yeast enolase 1 [20,21] was purified from recombinant yeast using ammonium sulphate precipitation and chromatographic purification on CM cellulose (Figure 2). The yeast enolase mutant of H159A was also purified using a similar protocol (data not shown). The purified proteins were tested for modifying activity on 3-phosphoglycerate, 3-phosphoglyceraldehdye, glucose and sucrose (Figure 3A).

### Chromatographic detection of sugars and intermediate metabolites

Paper chromatography was used for initial qualitative detection of sugars or sugar metabolites (Figure 3A) subsequently thin layer chromatography (Figure 3B) was used for detection and determination of modification as well as for measurements. Relative measurements of spot area with respect to the controls allowed determination of binding as described in methods.

### Binding constants

Binding constants for mutant proteins were determined and presented in Table 1. Dissociation constant (Kd) is a useful measure to describe the strength of binding or affinity between the proteins (P) and the ligands (L) and serves as an indicator as to how easy it is to separate the complex PL. With both PGDM and enolase mutants high micromolar (μM) concentration L is required to form PL indicating that the strength of bindings is rather moderate or even low. The smaller Kd, the stronger is the binding. The dissociation constant for 3-phosphoglycerate was found to be lower by both PGDM (655 ± 33 μm) and yeast enolase mutants S39A (676 ± 28 μm), H159A (797 ± 47 μm) compared to that for 3-phosphoglyceraldehde (822 ± 42, 835 ± 38 and 966 ± 31 μm respectively). The H159A had the weakest binding among other proteins for 3-phosphoglycerate. In qualitative measurements both proteins showed release or lack of binding when subjected to low pH (pH 4.0). In addition EDTA at concentrations that leads to chelation of Mg^2+ ^ions was very effective for release of ligands from enolase as well as from PGDM (Table 1).

### Reversibility in binding and repeated use

The PGDM showed binding with 3PGA and 3PGAL at pH 7.5 in presence of 50 mM NaCl. The binding was completely lost when the protein was exposed to pH 4.0 (Table 1). Binding was restored if the pH value was brought back to 7.5 within 30 min exposure to lower pH. The S39A mutant of yeast showed binding to 3PGAL as well as to 3PGA in presence of 10 mM NaCl and 10 mM MgCl_2_. Both 3PGA and 3PGAL failed to bind the protein and is released when subjected to 15 mM EDTA. The binding is restored upon removal of EDTA and addition of NaCl and MgCl_2_. Both proteins retained more than 50% initial binding upon repeated use, in this study we did not use more than 30 min incubation at lower pH during any single use. As shown in Table 2, the proteins retained qualitatively more than 50% binding for at least 8 cycles of repeated changes between pH 4.0 and pH 7.5. PGDM was less stable (8 cycles) than S39A or H159A mutants (10 cycles) each. This is important for reactor applications. While it is possible to control reactor operations where *in-situ *separation reactors can be bought back to pH immediately upon binding and for small reactors less than 30 min of residence time can be useful, more optimization studies are needed to establish stability at low pH for these proteins. For MgCl_2 _cycling, the proteins retained more than 85% activity even after 20 cycles and were not studied any further.

## Discussion

Microbes (as well as higher organisms) produce a number of binding and metabolizing proteins for sugar and other intermediate metabolic products of sugar metabolism pathways. Complex conversion necessitated using binding entities to enhance reactor performance as well as obtaining converted compounds in purified state.

In the biocatalytic CO_2 _fixation bioprocess, three modular reactors enables continuous fixation. The first module is Rubisco reactor where CO_2 _is fixed onto RuBP and converted into 3PGA, the second involves regeneration of ATP which acts as a cofactor for subsequent process and the third being RuBP regeneration [8,9]. Recently an added module has been devised for efficient capture of CO_2_, which is constructed before first module [10]. However, the generation of RuBP from converted 3PGAL requires a series of conversion in 11 reactors [8,9]. In many reactors in this series, the sugar or intermediate metabolites are generated that are not substrates for immediate subsequent reactors. Thus a specific capture and delivery will help eliminate dilution as well as interference in reactors where sugar intermediates are not direct substrate. Towards this goal, three different entities divided into biological and chemical moieties have potential for use. The biological entities include lectins or sugar binding proteins or non-immunogenic origin and inactive mutants of sugar binding enzymes. The chemical entities include the entities that recognize and binds aldehyde, ketones and alcohols. However, most of the biological entities provides high specificity, strong binding, reversible with respect to select physico-chemical conditions. They are also compatible with buffer systems with respect to conversion reactors in the loop where they are likely to be used. For 3PGAL and 3PGA as test sugars we have attempted using PGDM and inactive yeast enolase mutants (S39A, H159A) here. The determination of binding strength by these entities will enable their pilot tests in novel reactors in close loop with 3PGA to RuBP conversion reactors. It appears that the PGDM and enolase mutants are reversible binders and are stable with respect to repeated use-cycles. However, the binding is moderate at best (Kd values are in the range of 655–966 μM). Similar measurements for enolase provides 500 ± 28 and 673 ± 32 μM for binding with 3PGA and 3PGAL indicating that wild type enolase has slightly higher binding affinity for these metabolites. Further enhancement in binding by screening other different inactive enzyme mutants or that from different sources may help find more suitable entities.

## Conclusions

In the present report we demonstrate binding without chemical modification of 3PGA and 3PGAL by inactive yeast mutant enzymatic proteins PGDM and enolase. The binding seems to be specific as the proteins were not found to bind other sugars (glucose, fructose or sucrose in mixtures that were subjected to incubation). The binding is reversible with respect to pH and EDTA and proteins retain activity even after repeated use. The MgCl_2 _cycling seems to have less effect on protein stability with respect to binding and release and could be more suitable for use in larger reactors. While these criteria are suitable for use of the proteins for *in-situ *separation of sugar metabolites in reactors, but high micromolar dissociation constants (655–966 μM) suggest the moderate or even low binding strength. Finding inactive enzymes with high binding affinity or engineering them for this purpose will improve their utility.

## Methods

### Protein purification

Purified yeast PGDM and recombinant cultures for inactive yeast enolase mutants (S39A and H159A) were obtained from Dr. J. Nairn and Prof. J. M. Brewer as research gift respectively. The enolase mutants were purified following suitable modifications of published protocols [20,21]. Briefly, the overnight grown yeast cultures were harvested when absorbance values reached 12. The cells were harvested by centrifugation at 4000 × g and disrupted using sonication for 10 min with 20 sec gap and burst cycles and centrifuged at 12000 × g for 20 min. This crude protein solution was subjected to ammonium sulphate precipitation at 75% saturation. After ammonium sulphate precipiration the protein was dialyzed for 16 hours in a dialysis bag (molecular weight cutoff 3000) in 0.1 M Tris-Cl (pH8.5) containing 0.1 M NaCl and 0.1 mM MgCl_2_, with three changes in buffer solution. Enolase mutants were subjected to ion exchange chromatography for further purification on Acta prime protein purification system (Amersham Pharmacia Biotech, CA) using Q Sepharose column at a flow rate of 0.5 ml /minute at 4°C. Protein was eluted using a NaCl gradient (0.1 M to 0.3 M), about 95 fractions containing 8.6 ml each were collected. The proteins eluted in about conductivity equivalent of 0.1–0.15 M NaCl (fractions 5 to 9). This purified protein was temporarily stored at 4°C and used for subsequent experiments. Proteins were estimated using Bradford's method [22] using BSA (1 mg/ml) as standard. 10%SDS-PAGE was prepared and subjected to Coomassie blue staining.

### Paper and thin layer chromatography

Paper and thin layer chromatography was performed to demonstrate that the purified mutant proteins lacked any sugar modification capacity. Sugar mixtures in appropriate concentration were incubated with Proteins (PGDM or enolase mutants S39A and H159A) for up to 16 hours. At the end of incubation (every 2 hours), the mixtures were centrifuges and the aliquots from experimental mixtures were spotted onto the filter paper chromatograms (Whatman; 3 mm) or on TLC plates. The TLC analyses were performed on Plastic backed 20 cm × 20 cm Silica Gel 60 F254 plates with 0.2 mm layer thickness (Merck). After spotting with an applicator the samples were air-dried and placed in a TLC tank (27 cm × 24 cm × 7 cm) containing the solvent system. For both chromatography, the spots were air-dried and the chromatograms were dipped in the solvent system [60% v/v n-propanol/30% v/v conc.ammonia/10% v/v distilled water] and allowed to run for 5 hours. The chromatograms were removed from the solvent system and subjected to staining. Three different staining techniques were used to detect sugars, ammonium molybdate, silver nitrate and alpha-naphthol staining [23,24]. For ammonium molybdate staining, the paper chromatogram was dipped in the solution contianing: 5 ml 60 per cent w/w perchloric acid,10 ml and 0.1 N hydrochloric acid,25 ml, 0.4 per cent w/v ammonium molybdate, and made the volume to 100 ml with distilled water. The paper, after drying in a current of warm air for a few minutes to remove excess water, was subjected to heating at 85°C for 7 min in a water-jacketed oven. The spots in the chromatogram were also visualized using alkaline silver oxide reagent. This reagent was composed of two parts: first part containing 0.1 ml saturated aqueous silver nitrate plus 19 ml acetone and second part containing 0.5 g NaOH dissolved in 5 ml water and finally these two parts are added to 100 ml with ethanol. First part was mixed immediately before use and a few drops of water were added, with stirring, until all the AgNO_3 _is dissolved. The dried chromatogram was then dipped through the silver reagent and allowed to air dry for 10 min and subsequently dipped into ethanolic sodium hydroxide and again allowed to air dry. After the spots are visible, the paper was soaked in dilute (5 mg/l) sodium thiosulfate for 1 minute and rinsed in tap water. The last step dissolves the dark background and allows obtaining a permanent record. For staining with alphanapthol, paper chromatogram was dipped in the following solution: 1 per cent w/v alpha-napthol, 10 per cent v/v orthophosphoric acid and distilled water to make up the volume. The air-dried paper or TLC plate was heated for a few minutes at 85°C in a water-jacketed oven for color development.

### Determination of binding parameters

The dissociation constants for protein-sugar binding was estimated by measurements of area in chromatograms. For this purpose covalently immobilized protein A sepharose beads (Pharmacia Biotech, CA) was used. The proteins were immobilized on protein A using Amino link kit (Pierce Chemicals, CA). The known concentration of protein was incubated at room temperature (25°C) with varying concentration of sugar in the range of 1 μM to 1 mM in a 100 μl fixed volume. At the end of incubation (10 min), the mixture was centrifuged at 10000 × g and an aliquot of supernatant was spotted and chromatogram (TLC) was developed. A similar mixture but with BSA coupled beads served as control. Area calibration using varying concentration of sugar with a fixed aliquot spot volume was recorded under identical conditions. From the measurement of area in control and experimental set the free sugar was calculated such bound sugar is control minus sugar left in experimental set. The experimental data was used to draw a Scatchard type plot from where dissociation constant was calculated, represented by P as free protein, L as ligand and PL as the ligand-bound-protein, the dissociation constant is defined as Kd = [Pfree] [Lfree]/ [PL]. The dissociation constant Kd values for PGDM and S39A enolase for 3PGA and 3PGAL were calculated using experimental data using MS excel program.

## Authors' contributions

DB and DB carried out purification of enolase mutants, MK and SM carried out the binding assays. MTS, SC, AG and VG participated in design of the study and performing analyses, MTS also helped to draft the manuscript. SKB conceived of the study, and participated in its design and coordination and helped to draft the manuscript. All authors read and approved the final manuscript.

## Figures and Tables

**Table 1:** The binding constants of proteins for sugar metabolites.

**Protein**	**Condition**	**3-Phosphoglycerate**		**3-Phosphoglyceraldehyde**	
			**Kd (μm)**		**Kd (μm)**
PGDM	10 mM Tris-Cl pH 7.5, 50 mM NaCl	+++	655 ± 33	++	822 ± 42
	10 mM Tris-Cl pH 4.0, 50 mM NaCl	R		R	
Enolase, S39A	10 mM Tris-Cl pH 7.5, 10 mM NaCl, 10 mM MgCl_2_	+++	676 ± 28	++	835 ± 38
	10 mM Tris-Cl pH 7.5, 10 mM NaCl, 1 mM MgCl_2_, 15 mM EDTA	R		R	
Enolase, H159A	10 mM Tris-Cl pH 7.5, 10 mM NaCl, 10 mM MgCl_2_	++	796 ± 23	+	966 ± 31
	10 mM Tris-Cl pH 7.5, 10 mM NaCl, 1 mM MgCl_2_, 15 mM EDTA	R		R	

The binding constant was estimated from scatchard type plots using experimental data. The results provided here are from 3 replicates ± standard deviations. The substrates used in different concentration were analyzed with respect to BSA control for binding using TLC as described in methods. A silver nitrate protocol was used for detection and spot area determination. Plus signs denote the strength of ligandbinding; R indicates ligand release.

**Table 2:** The stability of proteins with use-cycles.

**Protein**	Number of Cycle when still active*
	
PGDM	8
	
Enolase, S39A	10
	
Enolase, H159A	10

* The retaintion of at least of 50% initial activity was considered as active. The cycle refers changes between pH 4.0 and 7.5 in 10 mM Tris buffer containing 50 mM NaCl. The values shown here are concurrent values from five independent experiments.
